# BNP signaling is crucial for embryonic stem cell proliferation

**DOI:** 10.1186/1471-2210-11-S1-O30

**Published:** 2011-08-01

**Authors:** Ikuo Tooyama, Essam M Abdelalim

**Affiliations:** 1 Molecular Neuroscience Research Center, Shiga University of Medical Science, Otsu, Shiga, 520-2192, Japan

## Background

Embryonic stem (ES) cells have the remarkable capacity to divide indefinitely while retaining their wide range differentiation potential, and they represent a promising source for cell transplantation therapies. Growth of ES cells requires a balance between survival, proliferation and self-renewal signals. The signaling pathways that regulate the proliferation of ES cells are of great interest. Brain natriuretic peptide (BNP), a member of natriuretic peptide family, is produced predominately in the heart, and recently, we have shown that BNP and its receptor (NPR-A, natriuretic peptide receptor-A) are expressed in ES cells and they play important roles in ES cell self-renewal [1].

## Methods and results

BNP and NPR-A were highly expressed in self-renewing murine ES cells, whereas the levels were markedly reduced after ES cell differentiation by the withdrawal of LIF. Targeting of BNP with short interfering RNA (siRNA) resulted in the inhibition of ES cell proliferation, as indicated by a marked reduction in the cell number and colony size, a significant reduction in DNA synthesis, and decreased numbers of cells in S phase. BNP knockdown in ES cells led to the up-regulation of gamma-aminobutyric acid receptor A (GABA_A_R) genes, and activation of phosphorylated histone (γ-H2AX), which negatively affects ES cell proliferation. In addition, knockdown of BNP increased the rate of apoptosis and reduced the expression of the transcription factor Ets-1. On the other hand, activation of endogenous GABA_A_R with muscimol (a GABA_A_R agonist) in low-density cultures significantly reduced the BNP mRNA and protein levels, which were associated with reductions in ES cell proliferation.

## Conclusion

Our data establish BNP as a novel regulator for the proliferation of murine ES cells. The present study defines a new pathway that is mediated by BNP-GABA_A_R for control ES cell propagation *in vitro*. These findings will facilitate our understanding of the signaling pathways that maintain the unusual proliferative characteristics of ES cells.
